# Supplementary material for: Predictive features of gene expression variation reveal mechanistic link with differential expression
Source: Mol Syst Biol. 2020 Aug 7;16(8):e9539. doi: 10.15252/msb.20209539 (PMC7411568; doi:10.15252/msb.20209539)
Supplement: Supplementary file 1 — Appendix [file MSB-16-e9539-s001.pdf]

Table of Contents

Appendix Figure S1……………………………………………………….2

Appendix Figure S2……………………………………………………….5

Appendix Figure S3……………………………………………………….7

Appendix Figure S4……………………………………………………….9

Appendix Figure S5……………………………………………………….11

Appendix Figure S6……………………………………………………….13

Appendix Figure S7……………………………………………………….15

Appendix Figure S8……………………………………………………….17

Appendix Figure S1. (a-b) Samples cluster by developmental time-points (a) and not by sequencing batches (b). First two principal components for 225 3' Tag-seq samples (75 samples at three time-points). Each dot represents a sample, samples are coloured by time-points (a) and sequencing date (b). All genes with quantified gene expression were used for the analysis. PCA was done on the raw expression counts after applying variance stabilization transformation from DESeq2 (Love *et al*, 2014). **(c)** Residual coefficient of variation (expression variation) reflects expression heterogeneity across samples at any given expression level. Gene expression across samples (y-axis, size factor normalized read counts) for genes binned by median expression level (20 subplots for 5%-percentiles by expression level) and ordered by expression variation (x-axis). Each dot represents a gene in one of 75 samples. Top and bottom 5% of genes by expression level were removed as potential source of outliers (the first and the last sub-plots, outlined with red). Data is shown for 10-12h time-point. **(d)** Gene expression variation is consistent across timepoints. Diagonal: distributions of gene expression variation values at three time-points. Upper triangular panels: Pearson correlation coefficients of gene expression variation between pairs of time-points. Lower triangular panels: scatter plots showing relationship between gene expression variation at different time-points (x and y-axis). Each dot represents a gene. Final set of 4074 genes was used at all three time-points. **(e)** Spearman correlation coefficients in expression variation (solid lines) and median expression levels (dashed lines) between pairs of genes located at varying quantile distances between their TSSs (actual distance intervals in kB are shown on the x-axis). Only gene pairs located on the same chromosome and with TSS-to-TSS distance <100 kB are considered. Spearman correlation coefficient (y-axis) and quantiles by distances (in kB) (x-axis) is plotted separately for gene pairs within the same TAD (blue) or split into different TADs (orange), TAD coordinates were taken from (Ramírez *et al*, 2018). Number of gene pairs in each group is indicated (blue and orange font for gene pairs within the same

TAD or crossing TAD border, respectively), only groups with >100 gene pairs are shown. **(f)** Proportion of gene expression variation explained by different components according to LIMIX variance decomposition (Methods). All three time-points (225 samples combined) were used in variance decomposition to achieve better convergence of the algorithm and more precise estimate of Cis component. Results for the final set of 4074 genes are plotted. Only Cis component (defined here as sum of Cis and (Cis x Environment) components from LIMIX) was used as a feature in subsequent random forest models.

Fig. S1

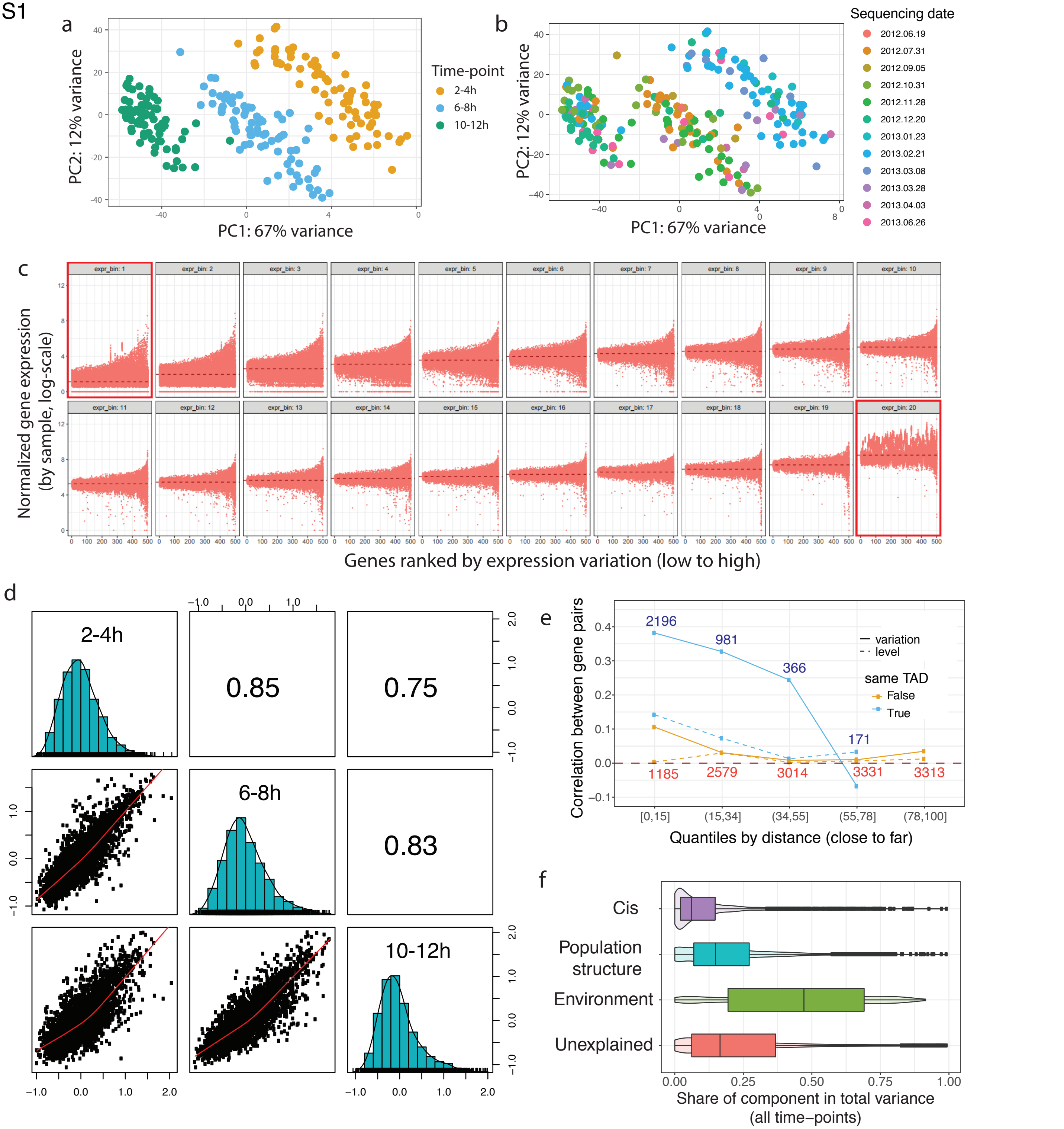
a
PC2: 12% variance
PC1: 67% variance
Time-point
2-4h
6-8h
10-12h
b
PC2: 12% variance
PC1: 67% variance
Sequencing date
2012.06.19
2012.07.31
2012.09.05
2012.10.31
2012.11.28
2012.12.20
2013.01.23
2013.02.21
2013.03.08
2013.03.28
2013.04.03
2013.06.26
c
Normalized gene expression
(by sample, log-scale)
Genes ranked by expression variation (low to high)
d
2-4h
0.85
0.75
6-8h
0.83
10-12h
e
Correlation between gene pairs
variation
level
same TAD
False
True
2196
981
366
171
1185
2579
3014
3331
3313
[0,15]
(15,34]
(34,55]
(55,78]
(78,100]
Quantiles by distance (close to far)
f
Cis
Population structure
Environment
Unexplained
Share of component in total variance
(all time−points)

Appendix Figure S 2. (a) Alternative measures of gene expression variation corrected for level dependence. From left to right: standard deviation after variance stabilizing transformation (*sd_vst*) from DEseq2 (Anders & Huber, 2010); residual median absolute deviation (resid_mad), inter-quartile range (resid_iqr), and residual standard deviation (resid_sd). The three later measures are LOESS residuals from regression on median expression level calculated in the same way as residual coefficient of variation (Methods). Data is shown for the final set of 4074 genes at 10-12h. Each dot represents a gene. Blue line shows LOESS regression fit, indicating no global dependence between corrected variation measures and median gene expression level. **(b)** Pearson correlation coefficient among different expression variation measures. Final variation measure used in the analysis is residual coefficient of variation (resid_cv). Correlations were computed on the final set of 4074 genes at 10-12h. **(c)** Random forest performance (R^2) for predicting expression variation based only on features (left, same as Fig 1d) or on features and median expression level (right). Data is presented as mean ± SD (5-fold cross validation). **(d)** Random forest performance (R^2) for alternative measures of expression variation (same as in a-b). Data is presented as mean ± SD (5-fold cross validation). Random forest was run on the set of features important for predicting expression variation (resid_cv, same as in Fig 1d). **(e)** Performance of random forest (R^2) for predicting expression variation for genes split into four quartiles by expression log2-fold change between 10-12h and 6-8h time-points. Data is presented as mean ± SD (5-fold cross validation). Numbers on the plot indicate number of genes in the corresponding quartiles. Horizontal red line indicates model performance on the full dataset (as in Fig 1d). **(f)** Performance of random forest for predicting expression variation when test and train sets come from different chromosome (arms). Prediction R^2 for each chromosome (arm) are shown as horizontal bars. Vertical red line indicates median performance across chromosomes.

Fig. S2

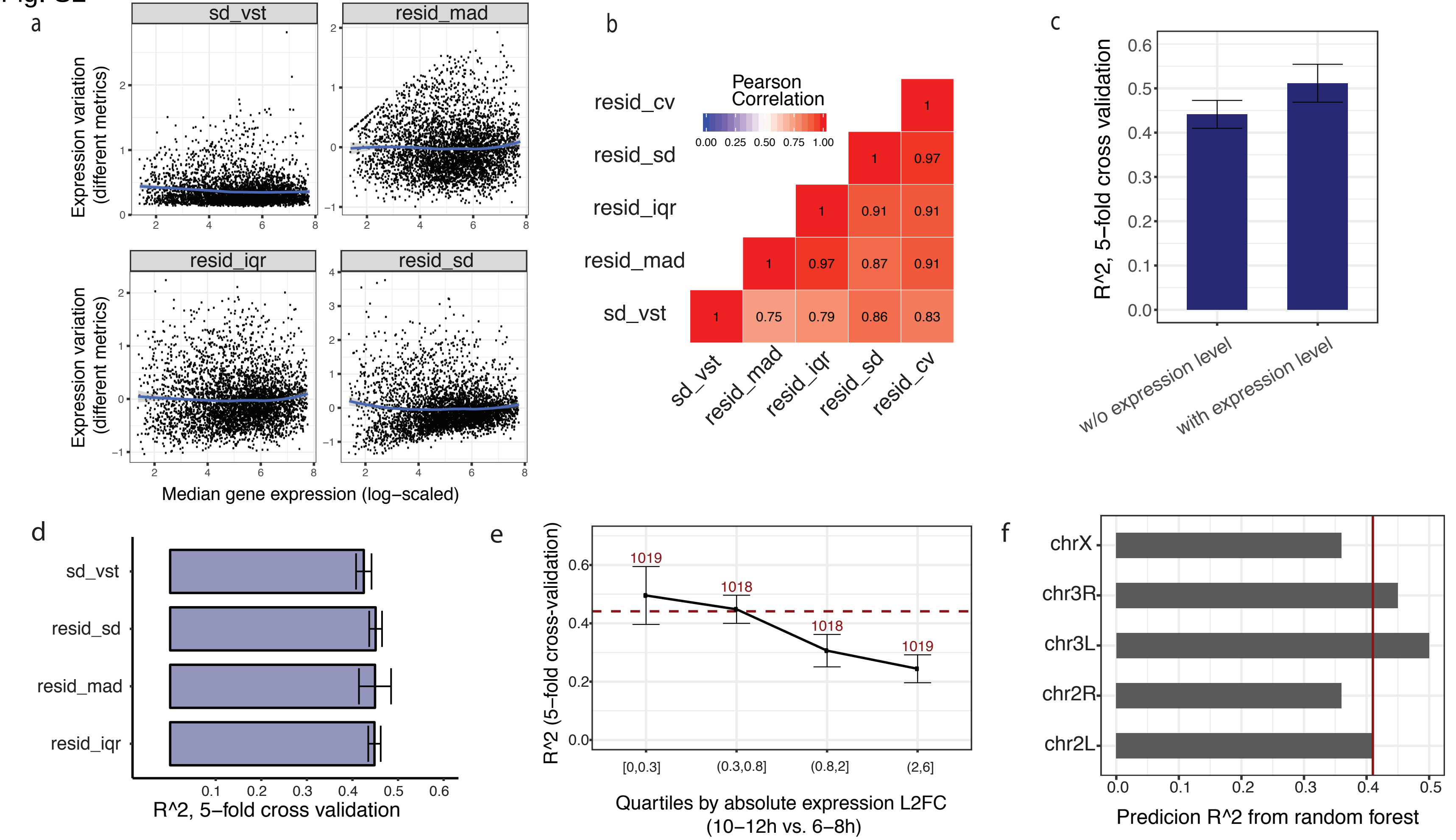
a
sd_vst
resid_mad
resid_iqr
resid_sd
Expression variation (different metrics)
Median gene expression (log−scaled)
b
Pearson Correlation
0.00 0.25 0.50 0.75 1.00
resid_cv
resid_sd
resid_iqr
resid_mad
sd_vst
1
1 0.97
1 0.91 0.91
1 0.97 0.87 0.91
1 0.75 0.79 0.86 0.83
sd_vst resid_mad resid_iqr resid_sd resid_cv
c
R^2, 5−fold cross validation
w/o expression level
with expression level
d
sd_vst
resid_sd
resid_mad
resid_iqr
R^2, 5−fold cross validation
e
R^2 (5−fold cross-validation)
1019
1018
1018
1019
[0,0.3]
(0.3,0.8]
(0.8,2]
(2,6]
Quartiles by absolute expression L2FC (10−12h vs. 6−8h)
f
chrX
chr3R
chr3L
chr2R
chr2L
Predicion R^2 from random forest

Supplementary Figure 3. (a) GO functional enrichment (Molecular function) of genes with broad (left panel) and narrow promoters (right panel) split into four quantiles by expression variation (x-axis). Top GO terms ranked by p-value are shown (full list in Dataset EV6). P-values (Benjamini-Hochberg correction) and gene ratio from compareCluster function (R clusterProfiler package) are reported. Quartiles of expression variation (1- lowest, 4 – highest, same as in Fig. 3c) were calculated for broad and narrow promoter genes separately. Quantile intervals for broad promoter genes (1 to 4): [-1.06,-0.444]; (-0.444,-0.266]; (-0.266,-0.0754]; (-0.0754,1.89]. Quantile intervals for narrow promoter genes (1 to 4): [-0.98,-0.173]; (-0.173,0.0751]; (0.0751,0.416]; (0.416,1.99]. (b) Regulatory plasticity (standard deviation across tissues and developmental time-points) of DHSs around TSSs of genes with different expression variation and promoter shape. Each DHS was assigned to the closest gene for this analysis, only DHSs located less than 500 bp. from the annotated TSSs are considered. Median of corresponding DHS standard deviations was calculated for each gene. Left panel: comparison of regulatory plasticity of broad and narrow promoter genes. P-values = Wilcoxon test. Number of genes in each group indicated. Right panel: DHS standard deviation (x-axis) plotted against gene expression variation (y-axis). Each dot represents a gene. Colours represent types of the corresponding genes. Distributions of DHS standard deviation of each group of genes are shown above the scatter plot. **(c)** Expression variation of different groups of genes (same as in b) depending on whether they have orthologs in human. Only orthologs with high conservation rank were considered (Methods). P-values = Wilcoxon test. Number of genes in each group indicated. Cohen's d: 0.39, 0.1, 0.92, and 0.47 for broad, narrow TFs, narrow TATA, and other narrow groups respectively.

Fig. S3

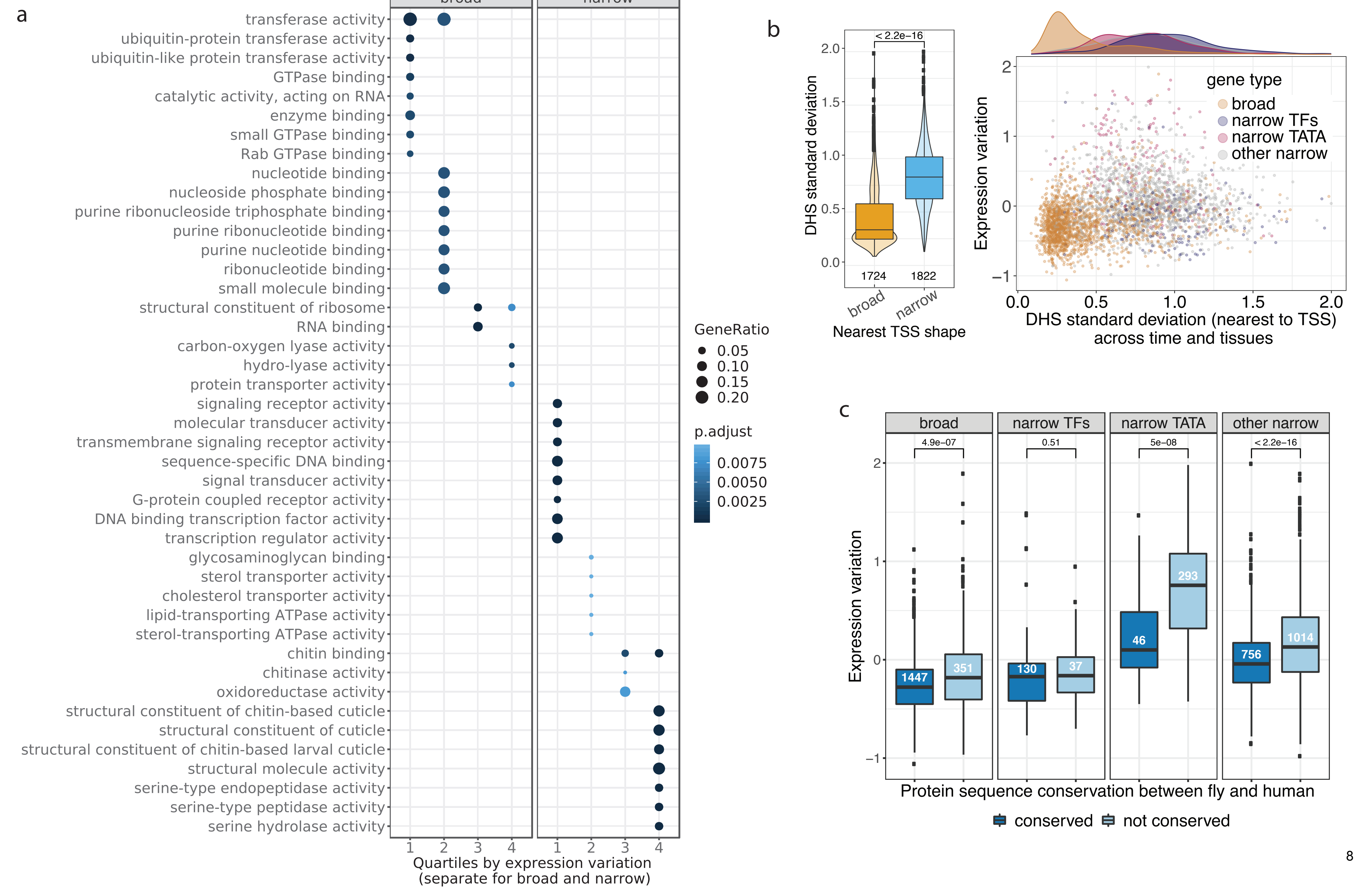
a
broad
narrow
transferase activity
ubiquitin-protein transferase activity
ubiquitin-like protein transferase activity
GTPase binding
catalytic activity, acting on RNA
enzyme binding
small GTPase binding
Rab GTPase binding
nucleotide binding
nucleoside phosphate binding
purine ribonucleoside triphosphate binding
purine ribonucleotide binding
purine nucleotide binding
ribonucleotide binding
small molecule binding
structural constituent of ribosome
RNA binding
carbon-oxygen lyase activity
hydro-lyase activity
protein transporter activity
signaling receptor activity
molecular transducer activity
transmembrane signaling receptor activity
sequence-specific DNA binding
signal transducer activity
G-protein coupled receptor activity
DNA binding transcription factor activity
transcription regulator activity
glycosaminoglycan binding
sterol transporter activity
cholesterol transporter activity
lipid-transporting ATPase activity
sterol-transporting ATPase activity
chitin binding
chitinase activity
oxidoreductase activity
structural constituent of chitin-based cuticle
structural constituent of cuticle
structural constituent of chitin-based larval cuticle
structural molecule activity
serine-type endopeptidase activity
serine-type peptidase activity
serine hydrolase activity
1 2 3 4 1 2 3 4
Quartiles by expression variation
(separate for broad and narrow)
GeneRatio
0.05
0.10
0.15
0.20
p.adjust
0.0075
0.0050
0.0025
b
< 2.2e−16
DHS standard deviation
2.0
1.5
1.0
0.5
0.0
1724
1822
broad
narrow
Nearest TSS shape
Expression variation
gene type
broad
narrow TFs
narrow TATA
other narrow
2
1
0
−1
0.0 0.5 1.0 1.5 2.0
DHS standard deviation (nearest to TSS)
across time and tissues
c
broad
narrow TFs
narrow TATA
other narrow
4.9e−07
0.51
5e−08
< 2.2e−16
Expression variation
1447
351
130
37
46
293
756
1014
Protein sequence conservation between fly and human
conserved
not conserved

Appendix Figure S4

(a-f). Differences between broad, narrow-low and narrow-high genes by the number of different TF motifs in TSS-proximal DHSs **(a,** Cohen's d=0.65 for broad vs. narrow-low, d=0.28 for narrow-low vs. narrow-high**)**, number of different TF peaks with motifs in TSS-proximal DHSs **(b,** Cohen's d=0.25 for broad vs. narrow-low, d=0.33 for narrow-low vs. narrow-high**)**, polymerase II pausing index **(c,** Cohen's d=0.64 for broad vs. narrow-low, d=0.30 for narrow-low vs. narrow-high**)**, number of different miRNA motifs in 3'UTR **(d,** Cohen's d=0.31 for broad vs. narrow-low, d=0.42 for narrow-low vs. narrow-high**)**, number of different RNA-binding protein motifs in 3'UTRs **(e,** Cohen's d=0.29 for broad vs. narrow-low, d=0.36 for narrow-low vs. narrow-high**)**, and number of TSS-distal DHSs (more than 500 bp and less than 10 kB around TSS) **(f,** Cohen's d=0.35 for broad vs. narrow-low, d=0.37 for narrow-low vs. narrow-high**)**. P-values from Wilcoxon rank test. **(g)** Top-50 important features for predicting expression variation within narrow promoter genes according to Boruta feature selection algorithm (with the corresponding importance for predicting expression level). Features are ordered by their importance for expression variation. Blue triangles indicate importance for variation, orange for level. Size and orientation of triangles correspond to absolute value and sign of correlation coefficient of feature with predicted variable, respectively. For binary features, point-biserial coefficient of correlation was used, otherwise Spearman coefficient of correlation. Label colours correspond to feature groups (same as in Fig. 2f). TFs enriched in TSS-proximal DHSs of broad and narrow promtoer genes (Fig 4c) are marked with red and blue start, respectively. **(h)** Gene scores by broad (left) and narrow (right) indices (same as Fig 4g). Colours correspond to gene types.

Fig. S4

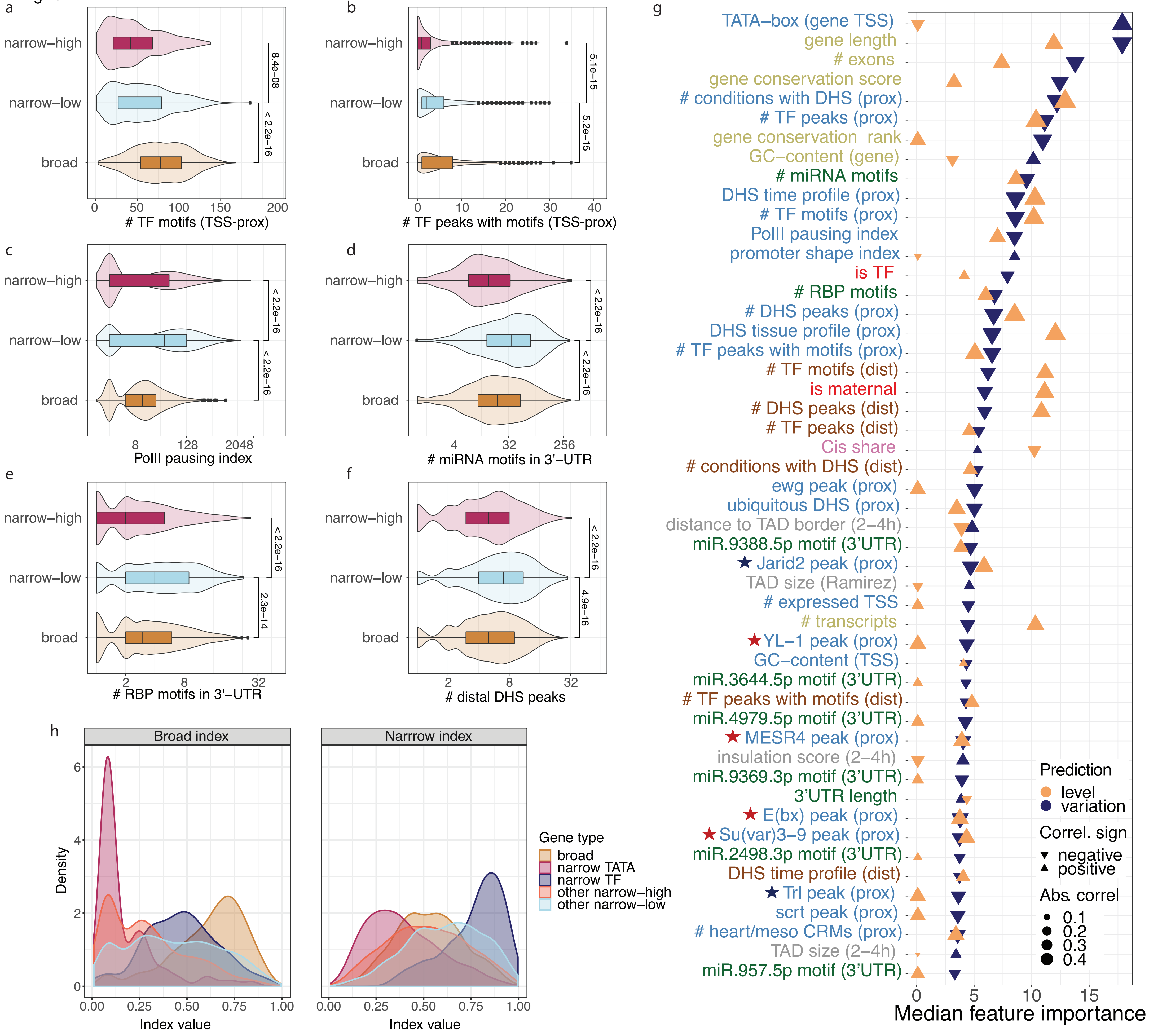
a
narrow−high
narrow−low
broad
8.4e−08
< 2.2e−16
0 50 100 150 200
TF motifs (TSS-prox)
b
5.1e−15
5.2e−15
0 10 20 30 40
TF peaks with motifs (TSS-prox)
c
< 2.2e−16
< 2.2e−16
8 128 2048
PolII pausing index
d
< 2.2e−16
< 2.2e−16
4 32 256
miRNA motifs in 3'−UTR
e
< 2.2e−16
2.3e−14
2 8 32
RBP motifs in 3'−UTR
f
< 2.2e−16
4.9e−16
2 8 32
distal DHS peaks
g
TATA−box (gene TSS)
gene length
exons
gene conservation score
conditions with DHS (prox)
TF peaks (prox)
gene conservation rank
GC−content (gene)
miRNA motifs
DHS time profile (prox)
TF motifs (prox)
PolII pausing index
promoter shape index
is TF
RBP motifs
DHS peaks (prox)
DHS tissue profile (prox)
TF peaks with motifs (prox)
TF motifs (dist)
is maternal
DHS peaks (dist)
TF peaks (dist)
Cis share
conditions with DHS (dist)
ewg peak (prox)
ubiquitous DHS (prox)
distance to TAD border (2−4h)
miR.9388.5p motif (3'UTR)
★ Jarid2 peak (prox)
TAD size (Ramirez)
expressed TSS
transcripts
★ YL−1 peak (prox)
GC−content (TSS)
miR.3644.5p motif (3'UTR)
TF peaks with motifs (dist)
miR.4979.5p motif (3'UTR)
★ MESR4 peak (prox)
insulation score (2−4h)
miR.9369.3p motif (3'UTR)
3'UTR length
★ E(bx) peak (prox)
★ Su(var)3−9 peak (prox)
miR.2498.3p motif (3'UTR)
DHS time profile (dist)
★ Trl peak (prox)
scrt peak (prox)
heart/meso CRMs (prox)
TAD size (2−4h)
miR.957.5p motif (3'UTR)
0 5 10 15
Median feature importance
Prediction
level
variation
Correl. sign
negative
positive
Abs. correl
0.1
0.2
0.3
0.4
h
Broad index
Narrrow index
Density
0 2 4 6
0.00 0.25 0.50 0.75 1.00
Index value
Gene type
broad
narrow TATA
narrow TF
other narrow−high
other narrow−low

Appendix Figure S5

(a) Expression variation (in our embryonic dataset) of genes differentially expressed (DE) upon any stress conditions in (Moskalev *et al*, 2015) compared to non-differentially expressed genes (non-DE). DE genes are split into 'up' (log-fold change > 0 in all stress conditions where gene is detected as DE), 'down' (log-fold change < 0 in all stress conditions where gene is detected as DE), and 'down-up' (gene is up-regulated in some experiments and down-regulated in the other) groups. Number of genes in each group is shown brackets in the figure legend. **(b)** Expression variation of genes differentially expressed (DE) upon different stress conditions from (Moskalev *et al*, 2015) compared to genes not differentially expressed in any of these experiments (non-DE). Stress conditions include: temperature (union of DE genes in three cold shock experiments at +4°C, 0°C, -4°C), radiation (union of 144 Gly, 360 Gly, and 864 Gly ionizing radiation), starvation (16 h), and fungi (union of 10 CFU and 100 CFU entomopathogenic fungus infection). P-values from Wilcoxon rank test, numbers under boxplots indicate number of genes. **(b-e)** Differences between DE genes (genes differentially expression in at least one stress condition from above) and non-DE genes by the number of conditions with DHS at TSS **(b),** and number of TSS-distal DHSs **(c),** polymerase II pausing index **(d)**, number of different miRNA motifs in 3' UTR **(e)** for broad and narrow promoter genes separately. P-values from Wilcoxon rank test, numbers under boxplots indicate number of genes. Cohen's d: (c) 0.22 and 0.57, (d) 0.15 and 0.34, (e) 0.17 and 0.32, (f) 0.69 and 0.73 for broad and narrow promoter genes, respectively. (**g-j**) Same as (c-f) for comparison between genes that were found as DE in more than 10 studies with genetic perturbation (Dataset EV8) vs. genes DE in 0-10 studies. Cohen's : (g) 1.02 and 0.43, (h) 0.14 and 0.28, (i) 0.14 and 0.07, (j) 0.02 and 0.47 for broad and narrow promoter genes, respectively.

Fig. S5

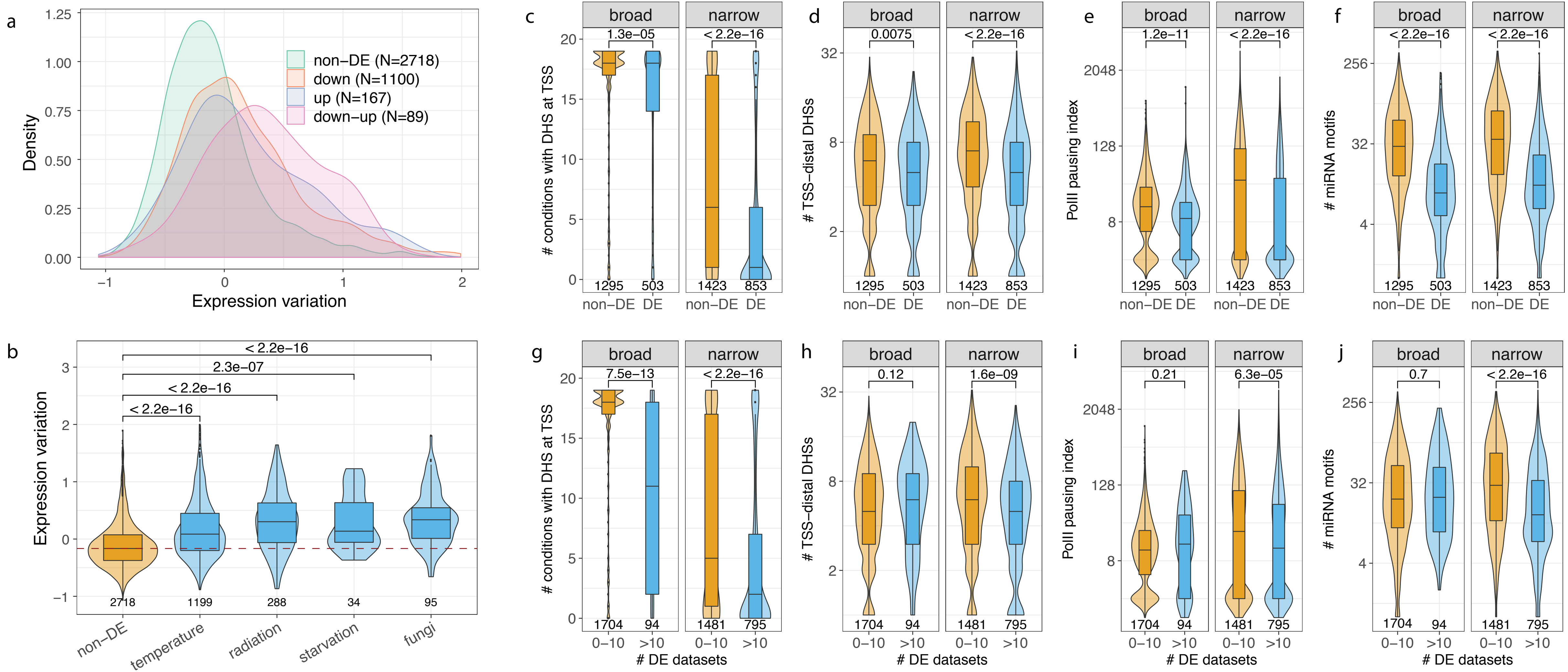
a
non–DE (N=2718)
down (N=1100)
up (N=167)
down–up (N=89)
Density
Expression variation
b
Expression variation
< 2.2e–16
2.3e–07
< 2.2e–16
< 2.2e–16
2718
1199
288
34
95
non–DE
temperature
radiation
starvation
fungi
c
conditions with DHS at TSS
broad
narrow
1.3e–05
< 2.2e–16
1295
503
1423
853
non–DE
DE
d
TSS–distal DHSs
0.0075
e
PolII pausing index
1.2e–11
f
miRNA motifs
g
7.5e–13
1704
94
1481
795
0–10
>10
DE datasets
h
0.12
1.6e–09
i
0.21
6.3e–05
j
0.7

Appendix Figure S6

Heatmap showing correlations (Spearman correlation coefficient) between expression levels and expression variations across different human tissues as well as their correlation with DE prior from (Crow *et al*, 2019). Labels contain tissue names from GTEx project; 'resid cv' stands for expression variation, 'median' indicates median expression level (log-transformed). 'Mean variation (by tissue adj)' is mean of expression variation across all tissues where a gene is expressed (corrected for expression level in each tissue separately, Methods). 'Mean variation (global adj)' is mean of coefficients of variations in all tissues where a gene was expressed, which was then corrected for mean expression level across the corresponding tissues (Methods). Colour code for the labels: red – expression levels; blue – expression variations; green – DE prior. Mean expression level and variation labels are highlighted with bold font.

Fig. S6

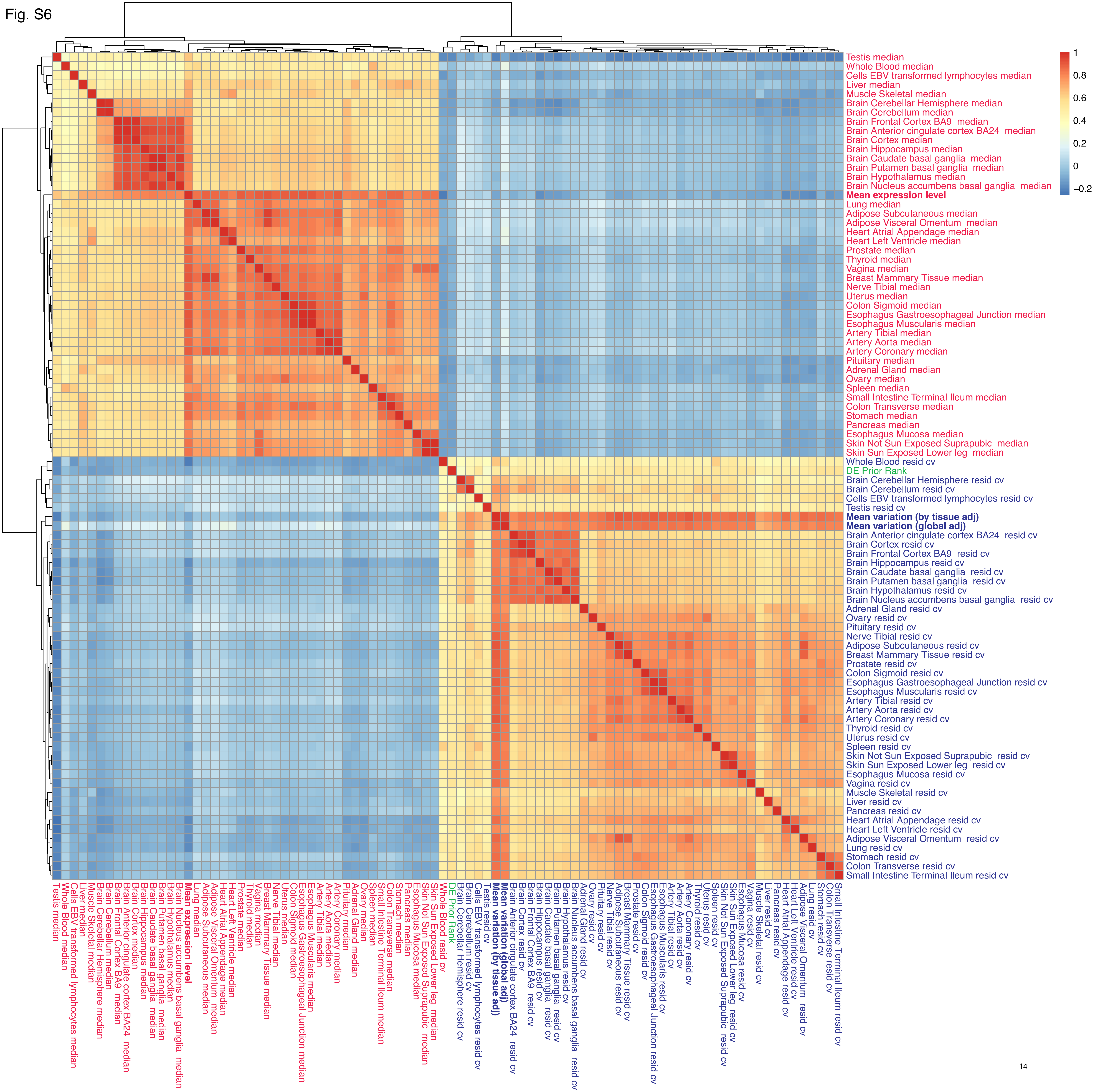
Testis median
Whole Blood median
Cells EBV transformed lymphocytes median
Liver median
Muscle Skeletal median
Brain Cerebellar Hemisphere median
Brain Cerebellum median
Brain Frontal Cortex BA9 median
Brain Anterior cingulate cortex BA24 median
Brain Cortex median
Brain Hippocampus median
Brain Caudate basal ganglia median
Brain Putamen basal ganglia median
Brain Hypothalamus median
Brain Nucleus accumbens basal ganglia median
Mean expression level
Lung median
Adipose Subcutaneous median
Adipose Visceral Omentum median
Heart Atrial Appendage median
Heart Left Ventricle median
Prostate median
Thyroid median
Vagina median
Breast Mammary Tissue median
Nerve Tibial median
Uterus median
Colon Sigmoid median
Esophagus Gastroesophageal Junction median
Esophagus Muscularis median
Artery Tibial median
Artery Aorta median
Artery Coronary median
Pituitary median
Adrenal Gland median
Ovary median
Spleen median
Small Intestine Terminal Ileum median
Colon Transverse median
Stomach median
Pancreas median
Esophagus Mucosa median
Skin Not Sun Exposed Suprapubic median
Skin Sun Exposed Lower leg median
Whole Blood resid cv
DE Prior Rank
Brain Cerebellar Hemisphere resid cv
Brain Cerebellum resid cv
Cells EBV transformed lymphocytes resid cv
Testis resid cv
Mean variation (by tissue adj)
Mean variation (global adj)
Brain Anterior cingulate cortex BA24 resid cv
Brain Cortex resid cv
Brain Frontal Cortex BA9 resid cv
Brain Hippocampus resid cv
Brain Caudate basal ganglia resid cv
Brain Putamen basal ganglia resid cv
Brain Hypothalamus resid cv
Brain Nucleus accumbens basal ganglia resid cv
Adrenal Gland resid cv
Ovary resid cv
Pituitary resid cv
Nerve Tibial resid cv
Adipose Subcutaneous resid cv
Breast Mammary Tissue resid cv
Prostate resid cv
Colon Sigmoid resid cv
Esophagus Gastroesophageal Junction resid cv
Esophagus Muscularis resid cv
Artery Tibial resid cv
Artery Aorta resid cv
Artery Coronary resid cv
Thyroid resid cv
Uterus resid cv
Spleen resid cv
Skin Not Sun Exposed Suprapubic resid cv
Skin Sun Exposed Lower leg resid cv
Esophagus Mucosa resid cv
Vagina resid cv
Muscle Skeletal resid cv
Liver resid cv
Pancreas resid cv
Heart Atrial Appendage resid cv
Heart Left Ventricle resid cv
Adipose Visceral Omentum resid cv
Lung resid cv
Stomach resid cv
Colon Transverse resid cv
Small Intestine Terminal Ileum resid cv
1
0.8
0.6
0.4
0.2
0
−0.2

Appendix Figure S7

(a-c) Distribution of promoter shape index and width by gene. **(a)** Histogram of genes' promoter shape index in *Drosophila* embryos (data from (Schor *et al*, 2017)). **(b)** Histogram of genes' promoter shape index in human lung tissue (Fantom5). **(c)** Histogram of genes' promoter width in human lung tissue (Fantom5). Density lines in **a-c** show fit of mixture distributions, vertical line indicates threshold for separating broad and narrow promoters (Methods). **(d)** Heatmap showing correlations (Spearman correlation coefficient) between promoter widths in different human tissues. Label names contain tissue names according to Fantom5 project.

Fig. S7

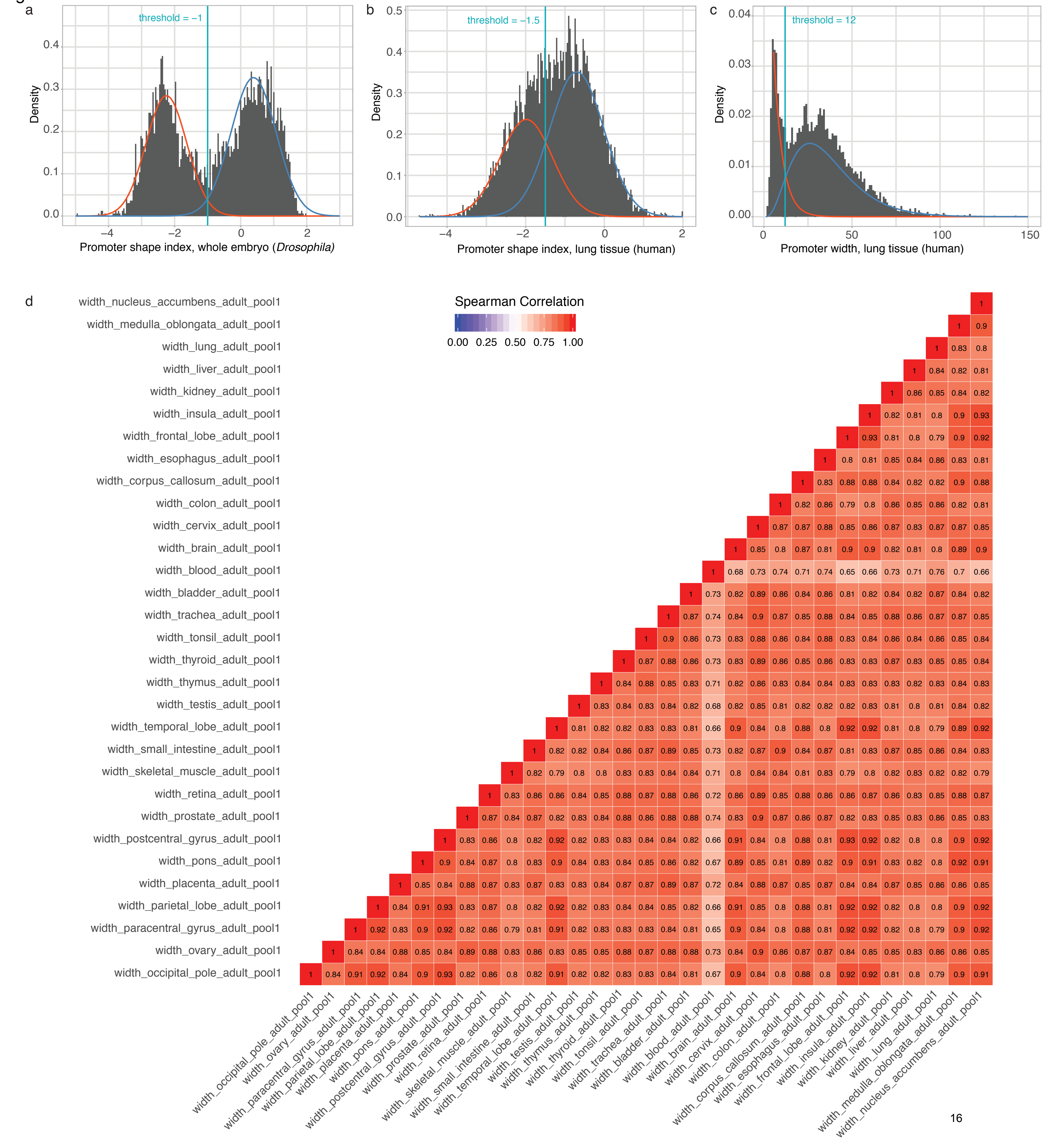
a
threshold = −1
Density
Promoter shape index, whole embryo (Drosophila)
b
threshold = −1.5
Density
Promoter shape index, lung tissue (human)
c
threshold = 12
Density
Promoter width, lung tissue (human)
d
Spearman Correlation
0.00 0.25 0.50 0.75 1.00
width_nucleus_accumbens_adult_pool1
width_medulla_oblongata_adult_pool1
width_lung_adult_pool1
width_liver_adult_pool1
width_kidney_adult_pool1
width_insula_adult_pool1
width_frontal_lobe_adult_pool1
width_esophagus_adult_pool1
width_corpus_callosum_adult_pool1
width_colon_adult_pool1
width_cervix_adult_pool1
width_brain_adult_pool1
width_blood_adult_pool1
width_bladder_adult_pool1
width_trachea_adult_pool1
width_tonsil_adult_pool1
width_thyroid_adult_pool1
width_thymus_adult_pool1
width_testis_adult_pool1
width_temporal_lobe_adult_pool1
width_small_intestine_adult_pool1
width_skeletal_muscle_adult_pool1
width_retina_adult_pool1
width_prostate_adult_pool1
width_postcentral_gyrus_adult_pool1
width_pons_adult_pool1
width_placenta_adult_pool1
width_parietal_lobe_adult_pool1
width_paracentral_gyrus_adult_pool1
width_ovary_adult_pool1
width_occipital_pole_adult_pool1

Appendix Figure S8

(a) Random forest performance (R^2) for predicting expression variation in lung dataset using different subsets of samples (using samples metadata from GTEx website). Data is presented as mean ± SD (5-fold cross-validation). **(b)** Spearman correlation coefficient between mean expression variation and TF features dependent on the width of the TSS-proximal region used to associate TFs with genes. Three intervals were considered: -/+500 bp around TSS (used in the main analysis for TFs and chromatin states), -300/+200 (used for some core promoter features, e.g. promoter shape and TATA-box), and -/+2 kB. Correlations are reposted for total number of TFs in the TSS-proximal region and top-10 important TFs for predicting mean expression variation in the main model (based on Boruta feature selection). **(c)** DE prior of specific genes groups (GWAS hits, essential genes, drug targets) compared to the distribution of DE prior for all genes in the dataset. Cohen's d: 0.22, 0.52, and 0.57 for GWAS catalog, Drug targets, and Essential genes respectively (comparison to All genes). P-values $< 2e-16$ for all comparisons to All genes (Wilcoxon rank test).

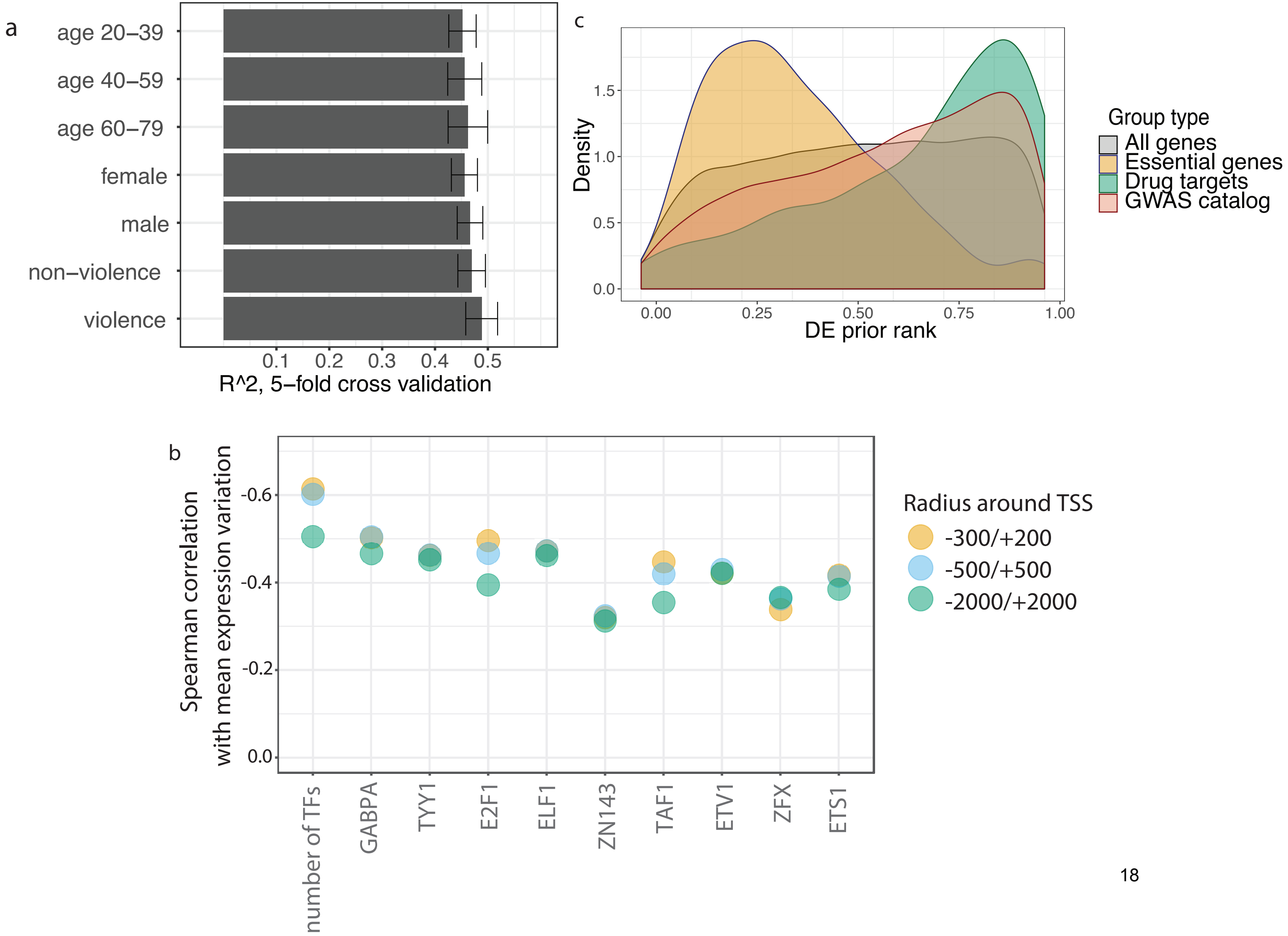

a
age 20–39
age 40–59
age 60–79
female
male
non–violence
violence
0.1 0.2 0.3 0.4 0.5
R^2, 5–fold cross validation
c
Density
1.5
1.0
0.5
0.0
0.00 0.25 0.50 0.75 1.00
DE prior rank
Group type
All genes
Essential genes
Drug targets
GWAS catalog
b
Spearman correlation
with mean expression variation
-0.6
-0.4
-0.2
0.0
number of TFs
GABPA
TYY1
E2F1
ELF1
ZN143
TAF1
ETV1
ZFX
ETS1
Radius around TSS
-300/+200
-500/+500
-2000/+2000
