# Supplementary material for: Predictive features of gene expression variation reveal mechanistic link with differential expression
Source: Mol Syst Biol. 2020 Aug 7;16(8):e9539. doi: 10.15252/msb.20209539 (PMC7411568; doi:10.15252/msb.20209539)
Supplement: Supplementary file 2 — Supplementary Datasets [file MSB-16-e9539-s002.zip › Datasets/Readme.docx]

**Dataset EV1.** Features used to predict expression level and variation in *Drosophila*. Columns:

Feature name – feature name as used in the master table (Supplementary tables 2 and 3)

Full name – full feature name

Class – feature class (see Table 1)

**Dataset EV2.** Final feature table including gene symbol and Flybase ID v6.13 (gene_name and gene_id), all features (before feature selection), median expression level (median), expression variation at 10-12h (resid_cv). Feature names are explained in Dataset EV1.

**Dataset EV3.** Full gene expression summary at three time-points. Columns:

gene_name and gene_id - gene symbol and Flybase ID v6.13

time – time-point when gene expression was measured (hours after fertilization)

median – median gene expression level (across 75 lines)

cv - expression coefficient of variation

resid_cv_extrRm, resid_cv_decrExprFilt, resid_cv_noDecrExprFilt_filtNA – expression variation at several intermediate filtering steps (removing extremes by expression level, genes that decreased in expression level between 10-12 and 2-4h. time-points, and genes with missing values in the feature table – see Methods)

resid_cv_decrExprFilt_filtNA - final expression variation values (same as resid_cv in Dataset EV2)

expr_l2fc_10vs2h, expr_l2fc_6vs2h, expr_l2fc_10vs6h – log2-fold change of gene median expression between the corresponding time-points

**Dataset EV4.** Feature importance scores (from Boruta) and correlations with predicted variables (Methods). Only features important in at least one prediction are included. NAs indicate non-significant features in the corresponding predictions.

Columns 1-3 are the same as in Dataset EV1. Columns 4-18:

med_imp_var - median feature importance for predicting expression variation

med_imp_med - median feature importance for predicting median expression level

med_imp_shape_ind - median feature importance for predicting promoter shape index

med_imp_broad_var -– median feature importance for predicting expression variation in broad promoter genes

med_imp_narrow_var - median feature importance for predicting expression variation in narrow promoter genes

med_imp_narrow_lev - median feature importance for predicting median expression level in narrow promoter genes

med_imp_broad_lev - median feature importance for predicting median expression level in broad promoter genes

cor_var - feature correlation with expression variation

cor_med - feature correlation with median expression level

cor_shape_ind - feature correlation with promoter shape index

cor_var_broad - feature correlation with expression variation in broad promoter genes

cor_var_narrow - feature correlation with expression variation in narrow promoter genes

cor_med_broad - feature correlation with feature correlation with expression variation in broad promoter genes

cor_med_narrow - feature correlation with feature correlation with expression variation in narrow promoter genes

**Dataset EV5.** Results of Fisher’s exact test for feature enrichments in specific groups of genes. Gene groups are defined in Fig. 3a. Feature names are explained in Dataset EV1. Columns 2-6 contain the values from the contingency tables used in the Fisher’s test. Odds ratios from the table are visualized in Fig. 4c. Columns:

comp – groups of genes being compared, e.g. broad promoter genes vs. narrow promoter genes (broad_vs_other) or ‘narrow-low’ genes vs. ‘narrow-high’ and ‘broad’ genes (narrow-low_vs_other).

Feature – feature for which the enrichment was calculated, e.g. presence of peaks for MESR4 in the TSS-proximal regions (modERN.MESR4.E0_24.prox)

num_group_feature – number of genes in the tested group having the feature

num_nongroup_feature - number of genes in the other group having the feature

num_group_nofeature - number of genes in the tested group not having the feature

num_nongroup_nofeature - number of genes in the other group not having the feature

pval - the p-value of the test

odds_ratio - an estimate of the odds ratio using conditional Maximum Likelihood Estimate (fisher.test function, R package *stats*)

pval_bh - the p-value after correction for multiple testing (Benjamini-Hochberg correction)

num_comp – number of comparisons, used in Benjamini-Hochberg correction. Enrichments of TFs (modERN.*tf_name*.prox) and gene categories were tested separately, hence number of comparisons differs.

**Dataset EV6.** Gene Ontology (GO) functional enrichment of genes grouped into eight clusters by promoter shape (broad/narrow) and expression variation (four quantiles by expression variation calculated separately within broad and narrow promoter genes). Exact values for quantiles are provided in Methods. Results are provided from compareCluster function (R package *clusterProfiler*). Columns:

Cluster - gene group tested for enrichment, e.g. 1.broad (bottom-25% by expression variation within broad promoter genes) or 4.narrow (top-25% by expression variation within broad narrow genes)

ID - GO category ID

Description – GO category description

GeneRatio – gene ratio

BgRatio – background ratio

pvalue - enrichment p-value

p.adjust – adjusted p-value (Benjamini-Hochberg correction)

qvalue - enrichment q-value

geneID - entrez IDs of genes from the GO category in the tested group

Count - number of genes from the GO category in the tested group

Ontology – Biological Process (BP) or Molecular Function (MF)

**Dataset EV7.** GO functional enrichment of genes grouped into four clusters by expression log2-fold change between 10-12h and 2-4h after fertilization (intervals are provided in column *Cluster*). Genes with log2-fold change below 0 were excluded from final analysis (Methods). Column names are similar to Dataset EV6.

**Dataset EV8.** Summary of the genetic perturbation studies used for the differential expression analysis. Columns:

Source – either Expression Atlas or in-house study (’lab’);

ID – dataset identifier for Expression Atlas, publication reference for in-house datasets;

Title - dataset name for Expression Atlas, publication reference for in-house datasets;

Date – date of submission to Expression Atlas, publication year for in-house datasets;

Factor – Experimental factor field from Expression Atlas, ‘genotype’ for in-house datasets; thres – log-fold change and p-value thresholds for calling differentially expressed (DE) genes;

N.genes – number of DE genes;

PMID - Pubmed ID of the publication;

cohen_d – Cohen’d d for comparing mean variation of DE vs. non-DE genes (cohen_d>0 indicates that DE genes are on average more variable);

mean_var_DE - mean variation of DE genes;

mean_var_non-DE – mean variation of non-DE genes;

wilcox_pval – p-value from Wilcoxon rank test;

n_genes_DE – number of DE genes in our dataset (out of 4074);

n_genes_non-DE – number of non-DE genes in our dataset (out of 4074).

**Dataset EV9.** List of GTEx tissues used in the analysis (RNA-seq data).

**Dataset EV10.** Tissue-specific expression data, DE prior (DE_Prior_Rank) and several gene annotations (essential genes, drug targets, GWAS hits; for details see Methods). Columns names for tissue-specific expression contain tissue names as listed in Dataset EV9. NA indicates that a gene is not expressed (or did not pass filtering criteria) in the corresponding tissue. Columns:

Gene_name – gene symbol;

Gene_id – Ensembl gene ID;

*Tissue_name*_mean – gene mean expression level (log-transformed) across individuals in the corresponding tissue;

*Tissue_name*_median - gene median expression level (log-transformed) across individuals

*Tissue_name*_cv - gene coefficient of variation across individuals

*Tissue_name*_recid_cv - gene expression variation across individuals (final measure of variation, adjusted for median dependence);

DE_Prior_Rank – Differential expression prior from (Crow et al. 2019);

GWAS_Upstream_gene_id and GWAS_Downstream_gene_id – EBI GWAS catalog genes (upstream or downstream of GWAS hits);

CEGv2_subset – essential genes;

Drug_targets_nelson and FDA_approved_drug_targets – drug targets.

**Dataset EV11.** Features used to predict expression level and variation in human. Columns:

Feature name – feature name as used in the master table (Supplementary tables 2 and 3)

Feature class – e.g. transcription factors or chromatin states

Feature type – tissue-specific (only used in Supplementary tables 13-15), non-tissue-specific or averaged across tissues (Methods)

**Dataset EV12.** List of Pantom5 tissues used in the analysis (CAGE data).

**Dataset EV13.** List of tissues with chromatin states used in the analysis (chromHMM)

**Dataset EV14.** Feature table for predicting expression variation (resid_cv) and median expression level (median, log-scale) across individuals in Lung tissue. Features used to predict expression variation and level are listed in Dataset EV10.

**Dataset EV15.** Feature table for predicting expression variation (resid_cv) and median expression level (median, log-scale) across individuals in Muscle tissue. Features used to predict expression variation and level are listed in Dataset EV10.

**Dataset EV16.** Feature table for predicting expression variation (resid_cv) and median expression level (median, log-scale) across individuals in Ovary tissue. Features used to predict expression variation and level are listed in Dataset EV10.

**Dataset EV17.** Feature table for predicting mean expression variation (mean_variation) and mean expression level (mean_median, log-scale) aggregated across all expressing tissues (Methods). Features used to predict expression variation and level are listed in Dataset EV10.

**Dataset EV18.** Feature importance scores (from Boruta) and correlations with predicted variables (Methods). Only features important in at least one prediction are included. NAs indicate non-significant features in the corresponding predictions.

Columns 1-3 are the same as in Dataset EV1. Columns 4-18:
